# Resetting the Stress System with a Mifepristone Challenge

**DOI:** 10.1007/s10571-018-0614-5

**Published:** 2018-09-01

**Authors:** Sergiu Dalm, Adriaan M. Karssen, Onno C. Meijer, Joseph K. Belanoff, E. Ronald de Kloet

**Affiliations:** 1 Division of Medical Pharmacology, Leiden/Amsterdam Center for Drug Research and Leiden University Medical Center, Leiden University, P.O. Box 9502, 2300 RA Leiden, The Netherlands; 2 Department of Medicine, Division of Endocrinology, Leiden University Medical Center, Room C-7-44, Postal zone C7-Q, PO Box 9600, Leiden, The Netherlands; 3 Corcept Therapeutics, Menlo Park, CA USA

**Keywords:** Stress, Brain, Behavior, RU38486, Glucocorticoid receptor, Mineralocorticoid receptor

## Abstract

Psychotic depression is characterized by elevated circulating cortisol, and high daily doses of the glucocorticoid/progesterone antagonist mifepristone for 1 week are required for significant improvement. Using a rodent model, we find that such high doses of mifepristone are needed because the antagonist is rapidly degraded and poorly penetrates the blood–brain barrier, but seems to facilitate the entry of cortisol. We also report that in male C57BL/6J mice, after a 7-day treatment with a high dose of mifepristone, basal blood corticosterone levels were similar to that of vehicle controls. This is surprising because after the first mifepristone challenge, corticosterone remained elevated for about 16 h, and then decreased towards vehicle control levels at 24 h. At that time, stress-induced corticosterone levels of the 1xMIF were sevenfold higher than the 7xMIF group, the latter response being twofold lower than controls. The 1xMIF mice showed behavioral hyperactivity during exploration of the circular hole board, while the 7xMIF mice rather engaged in serial search patterns. To explain this rapid *reset* of corticosterone secretion upon recurrent mifepristone administration, we suggest the following: (i) A rebound glucocorticoid feedback after cessation of mifepristone treatment. (ii) Glucocorticoid agonism in transrepression and recruitment of cell-specific coregulator cocktails. (iii) A more prominent role of brain MR function in control of stress circuit activity. An overview table of neuroendocrine MIF effects is provided. The data are of interest for understanding the mechanistic underpinning of stress system *reset* as treatment strategy for stress-related diseases.

## Introduction

Patients suffering from psychotic major depression benefit from a brief treatment with the glucocorticoid/progesterone receptor antagonist RU38486 or mifepristone (MIF), in a dose range of 600–1200 mg/day, once a day for four to seven days. This high dose of the antiglucocorticoid rapidly improves emotional expressions and cognitive abilities, and restores aberrant levels of the corticosteroids (Murphy et al. 1993; Belanoff et al. 2001, 2002; DeBattista and Belanoff 2006; Flores et al. 2006; Blasey et al. 2009, 2011; Block et al. 2018), see for meta-analysis (Garner et al. 2016). The fast amelioration of psychotic and depressive symptoms is thought to be at least in part due to restoration of glucocorticoid action to which the untreated patient with psychotic depression is resistant, while the anti-progestin activity of MIF is not implicated (Belanoff et al. 2001; Thomson and Craighead 2008). A recent analysis of all controlled phase 2 and 3 studies (MIF: *n* = 833 and placebo: *n* = 627) suggested that a dose of 1200 mg MIF/day for 7 days significantly reduced psychotic symptoms. For an effective treatment, circulating plasma levels of ≥ 1637 ng MIF/ml blood appeared required, while under such conditions basal HPA-axis activity was increased (Block et al. 2017, 2018). The data raise the question how MIF can exert this rapid effect on psychotic major depression.

In the current study, we will address this question from the perspective of adjustment in setpoint of stress system activity by the antiglucocorticoid (Ratka et al. 1988; Hu et al. 2012). Previous studies have shown that the effect of MIF on the HPA axis is dependent on the dose, route, and daily frequency of administration. In addition, a more prominent role of the brain mineralocorticoid receptors (MRs) after blockade of GR is likely, while also extrahypothalamic circuits are involved. Table 1 presents the published MIF effects on the HPA axis, which can be summarized as follows.

**Table 1:** Mifepristone effects on ACTH and corticosterone in rodents

Drug	Dose	Mode of administration	Outcome	Reference
Mifepristone	25 mg/kg rat	s.c. morning	No change in basal corticosterone for 4 h	Ratka et al. (1989)
Mifepristone	25 mg/kg rat	s.c. 60 min prior to stressor	Blunted peak and prolonged secretion of stress-induced corticosterone	Ratka et al. (1989)
Mifepristone	100 ng/rat	i.c.v. morning	No change in basal corticosterone for 4 h	Ratka et al. (1989)
Mifepristone	100 ng/rat	i.c.v. 15 min prior to stressor	Blunted peak and prolonged secretion of stress-induced corticosterone	Ratka et al. (1989)
Mifepristone	1, 3, 5, 10, 30, and 100 ng/rat	i.c.v. immediate after initial swim test	Doses ≥ 10 ng disinhibited HPA axis & interfered with retention immobility	de Kloet et al. (1988)
Mifepristone	100 ng infusion/rat	100 ng/h/for 3 days	Increased peak and decreased basal corticosterone levels	Van Haarst et al. (1996)
Mifepristone	100 ng/ rat	i.c.v	Increased basal ACTH and corticosterone levels at 1 h	Van Haarst et al. (1997)
Mifepristone	5 ng/ rat	bilateral dorsal hippocampus	Decreased basal ACTH and corticosterone levels at 1 h	Van Haarst et al. (1997)
Mifepristone	200 mg/ kg C57 mouse	orally via oats	Increased basal and stress-induced corticosterone	Dalm et al. (2008)
Mifepristone	30 mg/kg rat	s.c., 2 weeks daily	Suppressed basal and stress-induced HPA-axis activity, thymus weight reduced	Havel et al. (1996)
Mifepristone	10 mg/kg rat	s.c. 5 days, daily, 90 min after last injection	Suppressed basal and stress-induced HPA-axis activity	Wulsin et al. (2010)
C-108297, GR modulator	30 and 60 mg/kg rat	s.c. 5 days, daily, 90 min after last injection	Suppressed basal and stress-induced HPA-axis activity	Solomon et al. (2014)
C-118335 GR modulator/MR antagonist	30 and 60 mg/kg rat	s.c. 5 days, daily, 90 min after last injection	Suppressed basal and stress-induced HPA-axis activity	Nguyen et al. (2017), Nguyen et al. (2018)
Mifepristone	60 mg/kg C57 mouse, high-fat diet	orally, daily for 3 weeks in chow	Decreased basal am and pm (trend) corticosterone levels, decreased adrenal & thymus weight	van den Heuvel et al. (2016), Kroon et al. (2018)
C-108297, GR modulator	80 mg/ kg C57 mouse, high-fat diet	orally, daily for 3 weeks in chow	Decreased basal am and pm corticosterone level, decreased adrenal and thymus weight	van den Heuvel et al. (2016)
C-125281, selective GR antagonist	60 mg/ kg C57 mouse, high-fat diet	orally, daily for 3 weeks in chow	Restores high-fat disturbed HPA-axis activity, no effect on thymus and adrenals	Kroon et al. (2018)
Mifepristone	200 mg/ kg DBA mouse	oral infusion daily for 5 days, 2.5 h before amphetamine	Suppressed basal and amphetamine-induced corticosterone secretion	van der Veen et al. (2013)
Mifepristone	200 mg/kg mouse	oral infusion, twice a day for 4 days	Increased diabetes-induced ACTH and corticosterone levels, also in controls	Revsin et al. (2009)
Mifepristone	600 mg per human, male or female	daily for 8 days	Increased basal am and pm ACTH and cortisol, steeper slopes	Flores et al. (2006)
Mifepristone	600 mg/human (*n* = 5) vs control	daily for 5 days, chronic insomnia	2 weeks post-treatment. Decreased cortisol and ACTH/cortisol ratio 18.00–23.00 h; Increased ACTH and cortisol 23.00–7.00 h	Buckley et al. (2008)
Mifepristone	1200 mg/human, treatment effects on psychotic depression	daily for 7 days, measurement on day 7, 14, 28, 56	Basal ACTH and cortisol elevated at day 7 and 14 (*n* = 837, MIF). Strongest association of MIF with clinical efficacy beyond ACTH and cortisol	Block et al. (2017), Block et al. (2018)
MR antagonist	100 ng	i.c.v. and s.c	Increased basal and stress-induced corticosterone	Ratka et al. (1989)
MR antagonist	100 ng & 5 ng	i.c.v. & bilaterally dorsal hippocampus	Increased basal ACTH and corticosterone during both conditions	Van Haarst et al. (1997)

First, a central action of MIF is likely, since more than 100,000-fold lower dose centrally (10–100 ng local and i.c.v.) than systemically (10–200 mg s.c. /kg rat) could achieve feedback blockade in behaviorally active fashion (de Kloet et al. 1988; Ratka et al. 1989; van Haarst et al. 1997; Dalm et al. 2008). In spite of rapid degradation and poor brain penetration, such doses of MIF in the 10–200 mg range can enter the brain and do translocate the immunoreactive (ir) glucocorticoid receptor (GR) to the neuronal nuclei (Van Eekelen et al. 1987; de Kloet 1991). This finding was confirmed and further extended by the association of the MIF–GR complex to hippocampal Glucocorticoid Response Elements (GRE’s) (Spiga et al. 2011). ^3^H-MIF was found to bind to glucocorticoid receptor sites in human brain slices in vitro (Sarrieau et al. 1986).

Second, the prolonged and profound increase in secretion of corticosterone was the expected outcome of MIF interference with the stress-induced GR-mediated negative feedback (Ratka et al. 1989). The low GR occupancy during basal trough conditions explains the lack of acute MIF effects, but the antagonist can block during the p.m. phase or after minor stressors provided corticosterone levels are increased (Reul and de Kloet 1985).

Third, following MIF, the initial stress-induced rise in HPA-axis activity was attenuated. This blunted HPA-axis response likely was due to activation of co-localized hippocampal MRs. These MRs mediate rapid actions of corticosterone that precede those of GRs (de Kloet 1991; Joëls and de Kloet 1992; Karst et al. 2005; Joëls 2006). Indeed, central (hippocampal) MR blockade disinhibited the HPA-axis activity in rodents (Ratka et al. 1989; Oitzl et al. 1995; van Haarst et al. 1997) and humans (Dodt et al. 1993; Young et al. 1998; Deuschle et al. 1998).

Fourth, repeated daily administration for 5 days up to three weeks revealed an unexpected apparent GR agonism of MIF and blocked basal and stress-induced HPA-axis activity in rodents (Havel et al. 1996; Wulsin et al. 2010; van der Veen et al. 2013). Similar GR agonism rather than antagonism was noted with the novel selective GR modulators C-108297, C-125281, and the GR modulator/MR antagonist C-118335 (Solomon et al. 2014; Nguyen et al. 2017; Kroon et al. 2018; Van den Heuvel et al. 2016). Repeated MIF treatment caused a differential pattern of activation and inhibition of central inputs to the PVN, as judged from c-FOS activation (Wulsin et al. 2010).

Fifth, continuous infusion of 100 ng MIF/hr i.c.v. for 3 days enhanced stress-induced corticosterone secretion and increased the circadian amplitude of basal corticosterone level (Van Haarst et al. 1996; van Haarst et al. 1997). Continuous i.c.v. infusion improved spatial memory, which was impaired, however, when MIF was administered daily as a bolus of 100 ng/rat i.c.v. immediate before or after the learning trial (Oitzl and de Kloet 1992; Oitzl et al. 1998). The findings reinforce the notion of temporal and contextual diversity in glucocorticoid actions in various brain regions (Joëls and de Kloet 1992; Joëls et al. 2018).

Finally, twice rather than once a day MIF enhanced HPA-axis activity in diabetic rats (Revsin et al. 2009; Stranahan et al. 2008) and, if applied at the end of three weeks daily stress exposure, blocked the reduction in neurogenesis (Oomen et al. 2007; Mayer et al. 2006); even a single day treatment on day 18 was effective in this paradigm (Hu et al. 2012). MIF administered twice a day, at postnatal days 26–28, normalized deficits in hippocampal-dependent cognitive functions and associated neuronal activity that were previously induced by early-life maternal deprivation of rats (Arp et al. 2016; Loi et al. 2017).

A striking aspect of MIF’s efficacy is the ability *to reset* the stress system. This phenomenon was previously observed when the adrenals were rapidly (within 30 s under anesthesia) removed immediately following a severe stress to prevent the surge in glucocorticoid secretion. We noted a long-lasting (> 1 week) and profound potentiation in morphine- or β-endorphin-induced analgesia, which was correlated with increased opioid receptor binding and decreased hippocampal enkephalin and dynorphin mRNA expression. The antinociceptive effect of morphine was normalized if corticosterone was administered at the time of adrenalectomy (ADX) to mimic the stress or circadian rise. Also, 100 ng MIF i.c.v. administered during the p.m. circadian rise at 1 h prior to ADX caused long-term sensitization to morphine, apparently because the impact of the high prestress corticosterone in brain was antagonized (Ratka et al. 1988; Iglesias et al. 1991).

In the present study, we mimicked in mice the high-dose regimen of MIF that was beneficial in the patient studies (Block et al. 2018). For this purpose, we applied a non-invasive stress-free method for steroid delivery via oats (Dalm et al. 2008). After the first (1xMIF) and the seventh administration (7xMIF) we assessed (i) the circadian corticosterone secretion pattern; (ii) the behavioral and corticosterone response to novelty during exploration of a circular hole board at 24 h post-treatment. Hippocampal and hypothalamic MR, GR, and CRH mRNA expressions were also measured. Then, we investigated metabolism and brain penetration of MIF using Liquid chromatography–Mass Spectrometry–Mass Spectrometry (LC/MS/MS). Monolayers of pig kidney epithelial cells (LLC-PK1) stably expressing human multidrug-resistance P-glycoprotein (MDR1 Pgp) were used to examine the effect of MIF on cortisol transport (Karssen et al. 2001). We conclude with a discussion on the role of limbic-mesocortical circuits beyond HPA-axis regulation in prediction of treatment response to MIF.

## Methods

### Animals

Male C57BL/6J mice, 8–10 weeks of age, were purchased from Janvier (France). Upon arrival at the animal facilities (Gorlaeus Laboratory, LACDR, University of Leiden, The Netherlands), mice were single housed in a temperature (21 ± 1 °C) and humidity (55 ± 5%)-controlled room, with food and water *ad libitum;* for ten days before the start of the experiment (12–12-h light/dark cycle; lights on 0700–1900 h). During this period, mice were weighed and handled every other day. Experiments were approved by the Local Committee for Animal Health, Ethics and Research of the University of Leiden. Animal care was conducted in accordance with the European Union Directive 2010/63/EU.

### Study Design

The experiments were conducted with separate groups of mice. We measured (1) the 24-h circadian corticosterone secretion, following single and repeated administration of MIF (200 mg/kg; 1x/day for seven days). In addition, we collected blood samples around the time of the circadian corticosterone peak 32 h after the last administration of MIF. (2) Exploration behavior was measured in a circular hole board. Corticosterone concentrations were analyzed before (basal) and after exposure to the circular hole. (3) Following behavioral testing, mice were decapitated and brains were prepared for measuring the expression levels of MR, GR, and CRH mRNA in the hippocampus and paraventricular nucleus of the hypothalamus (PVN).

### Procedures

Familiarization of mice to oat administration and drug delivery procedures as described in (Dalm et al. 2008) are applicable to all experiments of this study.

### Familiarization to Oat Administration

One week prior to the start of the experiment, a feeding cup (2.3 cm diameter x 2.5 cm high) was taped to the floor in a corner of the home cage, opposite the nest location. For familiarization, three flakes of oats (Speltvlokken, Biologische teelt, Graanpletterij de Halm, Netherlands; ± 140 mg) were placed in the cup on three consecutive days every other day, 2 h after lights on. The top of the home cage was lifted and the sawdust was removed from the cup using an air puff generated with a pipette. Next, the oats were placed into the cup using forceps to minimize human odor transfer. Thereafter, the home cage was closed and the mouse was allowed to eat the oats undisturbed. All the oats were consumed within 10 min.

### Drug Delivery

Preparation of drug delivery via oats: One day prior to the experiment three flakes of oats were placed in a glass vial and the solutions containing GR antagonist or dissolvent (VEH) were applied. The glass vials containing the oats were kept at room temperature over night. Within 16 h, the solution was absorbed by the oats and they were dry when presented to the mice.

MIF (kindly provided by Corcept Therapeutics, Menlo Park, CA, U.S.A.) was dissolved in 1 ml 0.9% NaCl containing 0.25% carboxymethylcellulose and 0.2% Tween 20 (VEH = dissolvent). From this solution, 50 µl was applied to the oats (mice received a dose of 200 mg/kg MIF).

### Hormone Assays

The circadian corticosterone concentrations were measured in blood samples obtained via tail incision (Dalm et al. 2005). Briefly, a small incision with a razor blade at the base of the tail allowed collection of 50 µl blood within 90 s after opening of the animal’s cage. Following decapitation, trunk blood was collected individually in capillaries coated with potassium-EDTA (Sarstedt, Germany), stored on ice, and centrifuged with 13,000 rpm at 4 °C for 10 min. Plasma was stored at − 20 °C. Corticosterone concentrations were measured using commercially available radio immunoassay kits ^125^I-corticosterone (MP Biomedicals, Inc., NY, USA; sensitivity 3 ng/ml).

### Experiment 1: Effect of GR Antagonism on Corticosterone Secretion

#### Animals

Mice (*N* = 54) were randomly assigned to three treatment groups (*N* = 18 per group): (1) single mifepristone (1xMIF); (2) mifepristone once a day on seven consecutive days (7xMIF); or (3) VEH on seven consecutive days (VEH). Oats + MIF or Oats + VEH were placed in the feeding cup at 0900 h, and consumed within 10 min.

#### Experimental Design

The circadian corticosterone secretion was determined in blood samples collected via tail incision every two hour over a period of 24 h. The first blood sample was taken at 1100 h, i.e., two hours after MIF or VEH was administrated, and the last at 0900 h the next day. Subsequent blood samples were collected starting 32 h after the last administration around the circadian corticosterone peak at 1700, 1900, 2100, and 2300 h.

The three treatment groups (each *N* = 18) were divided in three subgroups each, consisting of six mice. Thus, from each mouse, one blood sample was taken every six hours and each time point consisted of six mice per group. During the dark period, blood sampling took place under red light conditions.

### Experiment 2: Corticosterone and Behavioral Responses to the Circular Hole Board

### Animals

Mice (*N* = 24) were randomly assigned to three treatment groups (*N* = 8 per group): (1) single mifepristone (1xMIF); (2) mifepristone once a day on seven consecutive days (7xMIF) or (3) VEH on seven consecutive days (VEH). Oats + MIF or Oats + VEH were placed in the feeding cup at 0900 h, and consumed within 10 min.

#### Experimental Design

Twenty-four hours after the last administration of MIF or VEH, we took a blood sample via tail incision, and placed the mouse for five min on the circular hole board; the behavioral response was analyzed. Immediately following behavioral testing, mice were decapitated. Corticosterone concentrations were determined in trunk blood. Brains were snap frozen in isopentane, pre-cooled on dry ice/ethanol, and stored at − 80 °C until further use, i.e., to determine, MR, GR, and CRH mRNA expression levels in brain tissue. Thymus and adrenals were removed and weighed.

#### Circular Hole Board

##### Apparatus

A gray round plate (Plexiglass; 110 cm diameter) with 12 holes (5 cm diameter, 5 cm deep) at equal distances from each other, and at a distance of 10 cm from the rim of the hole to the rim of the plate, was situated one meter above the floor in a different experimental room than the housing room. Light conditions on the surface of the board were 120 lx. To minimize and distribute odor cues, the surface was cleaned with 1%HAc and the board was turned (randomly clockwise and anticlockwise) before a mouse was tested. Behavior was recorded on videotape and analyzed with an automated tracking system (EthoVision 3.1, Noldus Information Technology, Wageningen, The Netherlands). The position of the mouse was sampled five times per second. To calculate the distance walked, we set the minimal distance between samples to 3 cm. The following parameters related to general activity, exploratory strategies, and possible anxiety-related behaviors were analyzed: distance walked (m) on the board and in specified zones: start center was defined as a circle of 30 cm diameter and the rim zone: a ring of 4.5 cm at the outer perimeter of the plate. Parameters: velocity (cm/s), number of holes visited; sequence of hole visits (*serial*: more than two holes in sequence; *perseveration*: repeatedly visiting the same hole or alternately visiting two neighboring holes); latency (s) to leave the center; latency (s) to and time spent (s) in rim zone.

#### In Situ Hybridization for MR, GR, and CRH mRNA

Brains were sectioned at − 20 °C in a cryostat microtome at 10 µm in the coronal plane through the level of the PVN and dorsal hippocampus. Sections were thaw-mounted on poly-L-lysine-coated slides (0.001%), air dried, and kept at − 80 °C until further use.

In situ hybridizations using ^35^S-labeled ribonucleotide probes (MR, GR, CRH) were performed as described previously (Schmidt et al. 2009). Briefly, sections were fixed in 4% paraformaldehyde and acetylated in 0.25% acetic anhydride in 0.1M triethanolamine/HCl. Subsequently, brain sections were dehydrated in increasing concentrations of ethanol. The antisense RNA probes were transcribed from linearized plasmids containing exon-2 of mouse MR and GR, and the full length coding regions of CRH (rat). Tissue sections (3–4 per slide) were saturated with 100 µl hybridization buffer containing 20 mM Tris–HCl (pH 7.4), 50% formamide, 300 mM NaCl, 1 mM EDTA (pH 8.0), 1x Denhardt’s, 250 µg/ml yeast transfer RNA, 250 µl/ml total RNA, 10 mg/ml salmon sperm DNA, 10% dextran sulfate, 100 mM dithiothreitol, 0.1% SDS, 0.1% sodium thiosulfate, and supplemented with approximately 1.5 × 10^6^ cpm ^35^S-labeled riboprobe. Brain sections were cover-slipped and incubated overnight at 55 °C. The next day sections were rinsed in 2xSSC, treated with RNaseA (20 mg/ml), and washed in increasingly stringent SSC solutions at room temperature. Finally, sections were washed in 0.1xSSC at 65 °C for 30 min and dehydrated through increasing concentrations of ethanol. All age groups were assayed together. Films were opposed to Kodak Biomax MR film (Eastman Kodak Co., Rochester, NY) and developed.

Autoradiographs were digitized, and optical density of the areas of interest was quantified using image analysis computer software (analySIS 3.1, Soft Imaging System GmbH). The average density of six measurements for each animal was calculated.

#### Statistics

Data are presented as mean ± SEM. The circadian profile of corticosterone was analyzed by analysis of variance (ANOVA) (factor: treatment) with repeated measurements, followed by Fisher’s Least Significant Difference (LSD) *post hoc* test. Total corticosterone (AUC: area under the curve) over 24 h was calculated for light and dark periods of 12 h, subjected to ANOVA, with treatment and time of the day as fixed factors. Statistical analysis was similar as for corticosterone. Body, adrenal, and thymus weights were analyzed using one-way ANOVA followed by Bonferroni’s multiple comparison *post hoc* test. Statistical significance was accepted at *p* < .05.

### Experiment 3: Metabolism and Membrane Transport of Mifepristone

Young adult male Wistar rats (Charles River, Germany) were housed under a 12/12-h light/dark cycle with lights on at 7:00 h in separate temperature (21 °C)- and humidity-controlled rooms. Food and drinking water were available *ad libitum*. Before and during experiments, rats were handled daily. Experiments were approved by the Local Committee for Animal Health, Ethics and Research of the University of Leiden. Animal care was conducted in accordance with the European Union Directive 2010/63/EU.

Rats were treated with MIF for five days. Body weight was monitored throughout this period. MIF suspended in an aqueous solution containing 0.25% carboxymethylcellulose/0.2% Tween 20 was administered by gavage at a dose of 50 mg/kg once a day. Groups treated with vehicle were also included in all experiments. On the last day, 1.5 or 3 h after the last injection, the animals were killed by decapitation at the end of the day during the circadian rise of corticosterone levels. Brain and plasma were collected and frozen until further use. Adrenals and thymus were also dissected and weighed. Plasma levels of corticosterone were determined using a ^125^I-corticosterone radioimmunoassay (MP Biomedicals, Costa Mesa, CA).

#### Corticosteroid Determination in Brain and Plasma

Using Liquid Chromatography–Mass Spectrometry–Mass Spectrometry (LC/MS/MS), steroid profiles were made of samples of the rat cortex and plasma. We measured levels of corticosterone, mifepristone (17β-hydroxy-11β-(4-dimethylaminophenyl)-17α-(1-propynyl)estra-4,9-dien-3-one), and its three main metabolites, the mono-demethylated (RU42633, 17β-hydroxy-11β-(4-monomethylaminophenyl)-17α-(1-propynyl)estra-4,9-dien-3-one), the didemethylated (RU42848, 17β-hydroxy-11β-(4-aminophenyl)-17α-(1-propynyl)estra-4,9-dien-3-one), and the hydroxylated (RU42698, 17β-hydroxy-11β-(4-dimethylaminophenyl)-17α-(1-propynol)estra-4,9-dien-3-one) (Deraedt et al. 1985). Samples were prepared for assay by dichloromethane/ethanol extraction essentially as previously described (Karssen et al. 2001).

The LC/MS/MS assays were performed on a Triple Stage Quadrupole mass spectrometer (Thermo Finnigan TSQ Quantum, San Jose, CA, USA) with an atmospheric pressure chemical ionization interface. A modification of the method of (van der Hoeven et al. 1997) was used. The analysis was performed in positive ionization mode using selective reaction monitoring of MIF, its three main metabolites, corticosterone, and dexamethasone. The [M + H]^+^ precursor ions were fragmented using argon as collision gas. The *m*/*z* ratios of the most abundant product ions were alternately scanned. The ion source temperature and the nebulization heater were kept at 200 °C and 400 °C, respectively. The voltages on the corona needle and on the electron multiplier were set at 10 µA. Each experiment, a new standard series was made in 25% methanol with concentrations ranging from 1 to 500 ng/ml of all steroids. Dexamethasone (1 µg/ml) was used as an internal standard. A Surveyor LC System (Thermo Finnigan) was used to inject 20 µl of the standard or extraction samples. A gradient of methanol–water (containing 1 g/l acetic acid) changing from 50/50% to 90/10% at a flow rate of 500 µl/min separated the steroids on an ADS C_18_ column. All samples were measured in duplo. The detection limit of this assay was 1–5 ng/ml for each steroid.

Steroid concentrations were calculated from a standard plot of area under the curve versus concentration. The standard curves usually displayed an r^2^ of more than 0.95. Presented data are corrected for recovery of dexamethasone, which was in the order of 25–50%.

#### Transepithelial Transport and Inhibition Studies

In order to examine the inhibitory action of MIF on Pgp-mediated cortisol transport, we used monolayers of the porcine kidney epithelial cell-line LLC-PK1, and LLC-PK1 cells stably transfected with cDNA of the human MDR1 gene encoding P-glycoprotein (LLC-PK1:MDR1) as previously described (Karssen et al. 2001). Cells obtained from the American Type Culture Collection (Manassas, VA) were kindly provided by the Dutch Cancer Institute (Amsterdam, The Netherlands) (Schinkel et al. 1995). MIF was added at a final concentration of 10 or 100 µM one hour before the addition of ^3^H-cortisol (Amersham Pharmacia Biotech, UK; specific activity 63 Ci/mmol) at a final concentration of 15 nM. In a separate experiment, a mix of MIF and metabolites at therapeutically relevant concentrations was added.

#### In Vivo Cortisol Uptake in Brain: Effect of MIF Pretreatment

To examine the in vivo effect of MIF on cortisol uptake into the brain, we have treated rats orally with 100 mg/kg MIF or vehicle (0.25% carboxymethylcellulose/0.2% Tween 20) (*N* = 7) each morning for 4 days. On the last day, all animals received a tracer dose of ^3^H-cortisol by s.c. injection 45 min after the last MIF treatment. After another 45 min, animals were decapitated. Trunk blood was collected and brain and liver were dissected. Tissue was weighed and solubilized in Soluene-350. Together with 100 µl plasma samples, tissue samples were counted to determine radioactivity.

### Statistical Analysis

Data were evaluated by Student’s *t* test or ANOVA followed by Tukey HSD post hoc test. The results of the monolayer experiments were analyzed by Repeated Measures ANOVA. Significance was taken at *p* < .05.

## Results

### Experiment 1: Effect of GR Antagonism on Corticosterone Secretion

#### Circadian Pattern of Plasma Corticosterone Level

**Fig. 1:**
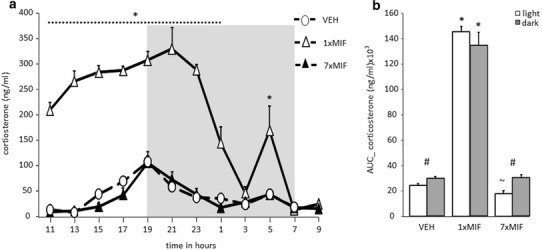
**a** Circadian secretion of corticosterone in ng/ml measured every 2 h in blood plasma of male mice C57BL/6J that received RU38486 (MIF) once (1xMIF) or for seven days (7xMIF). Mice were entrained in a 12–12-h light–dark cycle (dark phase from 1900 to 0700 h represented by the gray-shaded area). **b** Total corticosterone secretion in ng/ml during the light and dark period of the day, determined as area under the curve (AUC); ng/ml. Data are presented as mean ± SEM; *p* < .05 * versus other groups, ^#^ within groups, ^~^ 7xMIF versus VEH

Mice of all groups showed a circadian corticosterone rhythm (Fig. 1a; time *F* (11, 165) = 35.051; *p* < .001) as previously described (Dalm et al. 2005). The corticosterone secretion of control mice increased from 1500 h onwards, with peak levels (± 100 ng/ml) at the end of the light phase and the beginning of the dark phase (between 1700 and 2100 h). Interestingly, the frequency of MIF administration affected the course of the circadian rhythm (time*group: *F* (22, 165) = 15.992; *p* < .001). Corticosterone concentrations in 1xMIF-mice were significantly higher from 1100 until 0100 h (*p* < .01), reaching and maintaining peak levels from 1300 until 2300 h (± 300 ng/ml). Around 2300 h, concentrations readily declined until there was no difference in corticosterone concentration at 0300 h versus control and 7xMIF-administrated mice. There was a sudden significant increase versus controls (*p* = .001) and 7xMIF mice (*p* = .013), at 0500 h. In contrast, repeated MIF administration did not boost the concentrations of corticosterone as was observed for 1xMIF-administrated mice; the time course was similar to VEH mice. Overall, there was a main effect of treatment due to the high corticosterone concentrations in the 1xMIF mice (*F* (2, 15) = 550.923; *p* < .001).

#### Total amount corticosterone

The total amount of corticosterone calculated as area under the curve (AUC) over 24 h showed a main effect of treatment (Fig. 1b AUC: *F* (2, 17) = 392.094; *p* < .001). AUC corticosterone during the dark period (1900–0700 h) was higher than during the light period (0700–1900 h) in VEH and 7xMIF mice (paired t-test; both *p* < .01). 1xMIF mice had similar high AUC corticosterone levels during the light and dark periods, both significantly higher than VEH and 7xMIF mice. Interestingly, AUC corticosterone was lowest during the light period of 7xMIF mice (*p* < .039 versus VEH) due to the low corticosterone concentrations measured from 1500 till 1700 h.

#### Corticosterone Around the Circadian Peak: 32 h After Mifepristone Administration

**Fig. 2:**
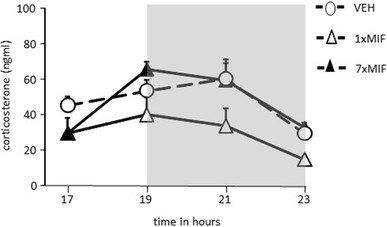
Corticosterone (ng/ml) secretion during the circadian peak in mice, 32 h after last administration of RU38486 (MIF), 1xMIF, 7xMIF, or VEH (dark phase from 1900 to 2300 h represented by the gray-shaded area). Data are presented as mean ± SEM

Treatment effects were found around the time of the circadian peak (Fig. 2, 1700–2300h; *F* (2, 15) = 6.308; *p* = .01). Thirty-two hours after the last administration, 1xMIF mice secreted less corticosterone than VEH (*p* = .007) and 7xMIF mice (*p* = .008). No statistical difference was found for corticosterone secretion patterns of VEH and 7xMIF groups.

### Experiment 2: Corticosterone and Behavioral Responses to the Circular Hole Board

#### Basal and Novelty-Induced Corticosterone Secretion

**Fig. 3:**
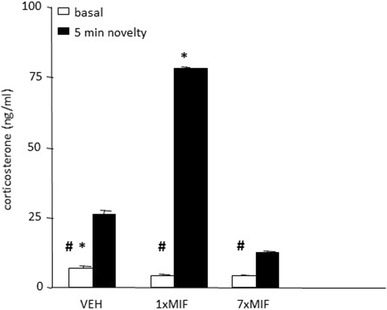
Basal and novelty (5 min exposure to the circular hole board)-induced corticosterone (ng/ml) were determined in mice, 24 h after last administration of VEH, 1xMIF, or 7xMIF. Data are presented as mean ± SEM; *p* < .05 * versus other groups, ^#^ within groups

Basal resting as well as novelty-induced corticosterone were affected 24 h after the last treatment (Fig. 3; treatment *F* (2, 44) = 17.175; *p* < .0001; time *F* (1, 44) = 45.980; *p* < .0001; treatment*time *F* (2, 44) = 17.626; *p* < .0001). Basal resting corticosterone differed significantly between the groups (*F* (2, 23) = 14.656; *p* < .001) and was lower in both MIF-treated groups than in VEH mice (*p* < .001). Basal corticosterone of 1xMIF and 7xMIF mice was comparable. After five min on the circular hole board, corticosterone was increased in all groups compared to baseline, however to a different degree (*F* (2, 23) = 19.074; *p* < .0001). Corticosterone levels in 1xMIF were 300% of the VEH group and 700% of the 7xMIF group (both *p* < .0001); corticosterone of the VEH group was about twice as high as in the 7xMIF group (*p* < .05).

#### Expression of MR, GR, CRH mRNA in Hippocampus and PVN

**Fig. 4:**
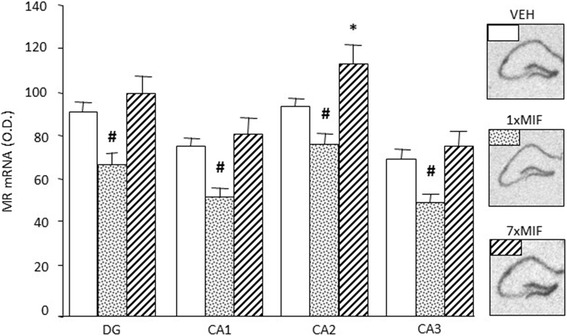
Expression of MR mRNA, measured as optical density (OD) in the hippocampal subfields dentate gyrus (DG), CA1, CA2, and CA3, 24 h after last administration of VEH, 1xMIF, or 7xMIF. Data are presented as mean ± SEM; *p* < .05 * versus other groups, ^#^ within groups

Hippocampal MR mRNA expression was differentially affected by treatment, 24 h post-administration, across all subfields (Fig. 4; treatment – DG: *F* (2, 23) = 11.005; *p* = .001; CA1: *F* (2, 23) = 12.887; *p* = .001; CA2: *F* (2, 23) = 14.267, *p* = .001; CA3: *F* (2, 23) = 11.550; *p* = .001). MR mRNA expression was reduced across all subfields in 1xMIF-mice compared to VEH and 7xMIF-mice (*p* < .05). Repeated MIF administration increased MR mRNA expression in the CA2 specifically versus VEH and 1xMIF-mice (*p* = .016 and *p* = .001, respectively). Neither GR nor CRH mRNA expression in hippocampus and PVN were affected by treatment (data not shown).

#### Exploration on the Circular Hole Board

Twenty-four hours after administration, the behavioral response differed during five min exploration on the circular hole board (Table 2: MANOVA: *F* (20, 26) = 3.772; *p* = .001). Following initial slower movement out of the central start position, 1xMIF mice showed hyperactivity: they walked longer distances, with a faster speed of moving, visited more holes, and made more rim dips (vs. VEH and 7xMIF-mice: *p* < .05). Interestingly, 7xMIF-mice made more use of a serial search strategy (vs. VEH mice: *p* = .05).

**Table 2:** The behavioral response during five min circular hole board exposure, 24 h after the last administration with RU38486 (MIF)

	VEH	1xMIF	7xMIF
General activity			
Distance walked (m)	7.9 ± 0.7	*15.0* ± 1.9*	7.5 ± 0.9
Speed of moving (cm/s)	8.6 ± 0.4	*11.6* ± 0.8*	9.9 ± 0.3
Total hole visits	14.8 ± 2.1	*24.8* ± 1.8*	17.0 ± 2.4
Search strategy			
Latency (s) from center	8.4 ± 1.4	*14.0* ± *1.4*^*#*^	11.8 ± 2.1
Latency (s) first hole visit	13.9 ± 0.8	16.0 ± 2.6	*18.4* ± *0.7*^*#*^
% Serial	16.5 ± 5.2	28.2 ± 3.6	36.6 ± 10.2^*#*^
% Perseveration	48.6 ± 5.8	39.3 ± 5.2	52.1 ± 5.1
Anxiety related			
Latency (s) to rim	63.0 ± 13.1	55.1 ± 12.4	69.9 ± 8.5
Number of rim dips	12.8 ± 1.4	*18.4* ± 2.0*	11.1 ± 1.2
Number of boli	1.1 ± 0.7	0.8 ± 0.4	1.3 ± 0.8

Data are presented as mean ± S.E.M.; *p* < .05 * versus other groups; # versus VEH

*Bold italic* indicates significant differences

#### Other Physiological Measures

Treatment did not influence body weight. Adrenal weight (*F* (3, 34) = 3.733; *p* = .035) was highest in both MIF groups, but significantly higher in 7xMIF than in VEH (*p* = .005): adrenals in mg, mean ± SEM: VEH 23.5 ± 2.8; 1xMIF 31.3 ± 4.3; 7xMIF 39.3 ± 2.9. Thymus weight was lower in both MIF groups, but passed statistical significance (*F* (3, 34) = 3.100; *p* = .059): thymus in mg, mean ± SEM: VEH 411.0 ± 38.9; 1xMIF 371.5 ± 15.1; 7xMIF 321.1 ± 23.6.

### Experiment 3: Metabolism and Membrane Transport of Mifepristone

#### In Vivo Steroid Uptake in Brain

**Fig. 5:**
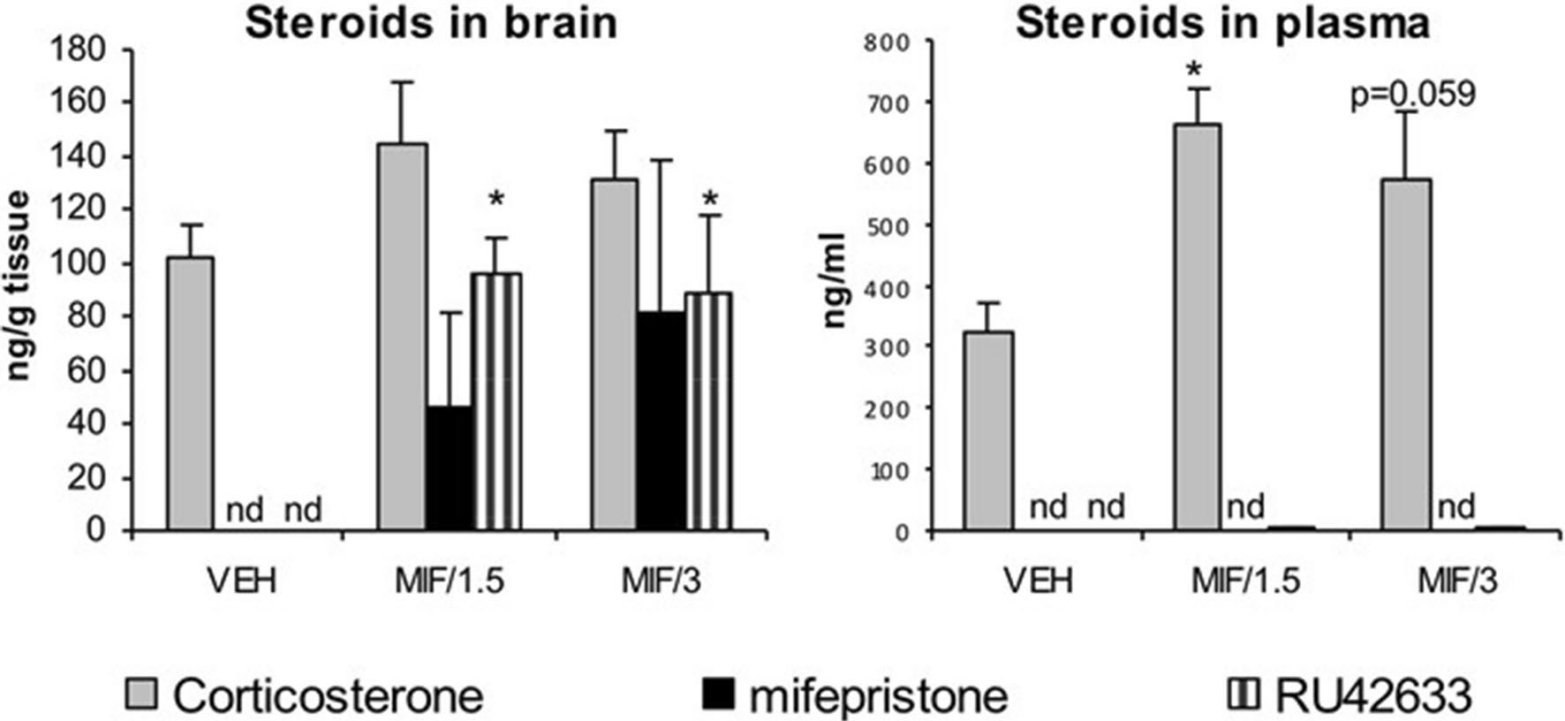
Steroid levels at 1.5 or 3 h after the last oral administration of 50 mg/kg mifepristone; levels of mifepristone were undetectable in plasma, but clearly detectable in brain although with high variability. Brain RU42633 levels were significantly higher in MIF-treated animals compared to vehicle treated rats (*F*_(2,15)_ = 13.12, *p* < .01). Corticosterone levels were significantly higher in plasma but not in brain of rats treated with MIF (*F*_(2,15)_ = 7.94, *p* < .01) compared to vehicle -treated rats. The concentrations of RU42848 and RU42698 were below the detection limit in both plasma and brain. *N* = 4–6, shown is mean + sem, *p < .05, Tukey post hoc test. Note the difference in scale

In experiment 1, the steroid profiles of rat brain and plasma made with LC/MS/MS showed that after oral treatment with 50 mg/kg/day, MIF could not be detected in plasma at either 1.5 or 3 h after the fifth and last treatment. Neither could any of its metabolites. In contrast, MIF was detectable in the brain with no significant difference between both time points, although individual variability in brain MIF levels was quite high. Comparable but more consistent levels were found for RU42633 in brain after treatment with MIF (Fig. 5). The mono-demethylated metabolite was also present at very low, but detectable levels in plasma. Moreover, plasma levels significantly correlated with brain levels for MIF-treated animals (*r*^2^ = 0.52, *p* < .01). Corticosterone levels in brain also correlated strongly with plasma levels (*r*^2^ = 0.48, *p* < .01) as determined with LC/MS/MS, and the latter values were validated with ^125^I-corticosterone radioimmunoassay (*r*^2^ = 0.89, *p* < .01; data not shown). After oral treatment with MIF, corticosterone levels increased significantly in plasma (*F* (2,15) = 7.94, *p* < .01) (Fig. 5). In brain, the increase did not reach statistical significance. MIF treatment did not affect adrenal or thymus weight nor body weight.

#### Inhibition of Cortisol Transport In Vitro

**Fig. 6:**
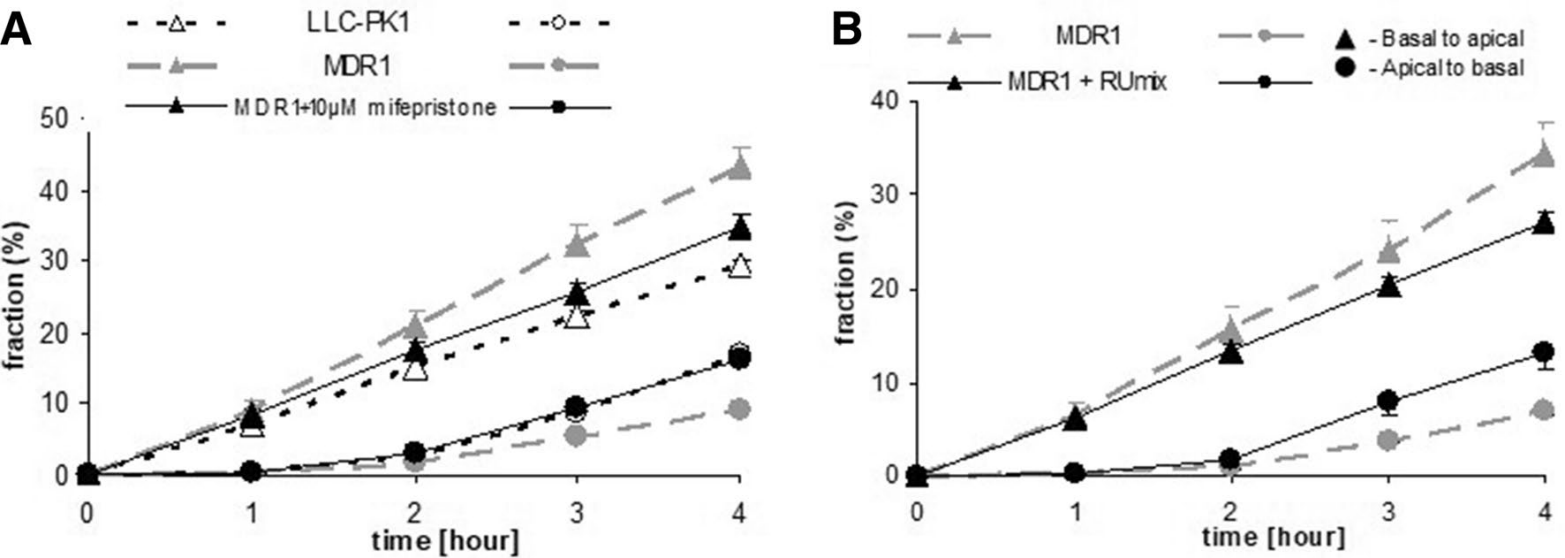
**a** Fraction of activity of ^3^H-cortisol present in medium at different time points after adding 15 nM ^3^H-cortisol to the opposite compartment at *t* = 0 in absence or presence of MIF. Transepithelial transport from basal to apical compartment and vice versa was measured in MDR1-transfected LLC-PK1 monolayers. Repeated measures ANOVA showed a significant time * cell type * MIF * direction of transport interaction (*p* < .01). In the presence of 10 µM MIF, transport of ^3^H-cortisol in monolayers of MDR1-transfected cells is inhibited and not different from transport of cortisol in monolayers of hosts cells. Data are presented as mean ± sem of three wells. MIF did not affect cortisol transport in untransfected monolayers (data not shown). **b** A mix of MIF and its three main metabolites at therapeutically relevant concentrations(see text) inhibits the transport of ^3^H-cortisol in MDR1-transfected monolayers

The in vitro experiments confirmed our previous observations (Karssen et al. 2001) that in MDR1-monolayers, cortisol was transported in a highly polarized fashion (Fig. 6a). In presence of 10 µM MIF, this transport was inhibited. ANOVA followed by post hoc analysis shows that at *t* = 4 in presence of MIF, MDR1-transfected monolayers were not different from untransfected monolayers with regard to transport of ^3^H-cortisol, while both were statistically different from the untreated MDR1 monolayers. 100 µM MIF was not able to further enhance the inhibitory action on cortisol transport (data not shown).

As the three main metabolites of MIF are structurally closely related to MIF, they may inhibit Pgp as well. Therefore, we tested the ability to inhibit cortisol transport of a mix of MIF and metabolites at therapeutically relevant concentrations (Lähteenmäki et al. 1987). A mix of MIF, RU42848, RU42698 (2.5 µM each), and 6 µM RU42633 affected cortisol transport in MDR1 monolayers to a similar extent as 10 µM MIF alone (Fig. 6b), whereas 2.5 µM MIF alone inhibited transport to a minor extent only (data not shown).

#### In Vivo Uptake of ^3^H-Cortisol: Effect of Mifepristone Pretreatment

In a preliminary experiment, at 45 min after ^3^H-cortisol, blood plasma radioactivity was Veh (*n* = 7) versus MIF (*n* = 8), 0.88 versus 0.65 nCi/ml, while in brain, Veh versus MIF was 0.08 versus 0.11 nCi/mg tissue. Although the amount of radioactivity in MIF-treated animals was not significantly enhanced in any tissue or plasma, the blood/brain ratio of radioactivity administered as ^3^H-cortisol was significantly increased in MIF-treated animals, indicating a facilitation of cortisol uptake into the brain. The fold increase (1.9x) is less than in Pgp knock-out mice as previously determined (3.5x) (Karssen et al. 2001).

## Discussion

The current data extend in male mice and rats the notion that a single challenge with MIF interferes with glucocorticoid feedback causing a long-lasting elevation of corticosterone secretion (Ratka et al. 1989; Dalm et al. 2008). Moreover, we found that at 24 h after ingestion of MIF, stress responsivity is profoundly enhanced. However, upon repeated high-dose MIF (200 mg/kg) daily administration, the circadian- and stress-induced HPA-axis activity are abolished. This apparent GR agonism of MIF upon repeated administration was previously also reported (Havel et al. 1996; Wulsin et al. 2010; van der Veen et al. 2013; Solomon et al. 2014; van den Heuvel et al. 2016; Nguyen et al. 2017, 2018; Kroon et al. 2018); however, with the lower doses in these studies, stress responsivity was attenuated rather than abolished (see also Fig. 5). At the same time, the weight of the adrenals was increased in our study. Adrenal weight was not altered with the lower 20-fold lower dose of MIF over 5 days (Wulsin et al. 2010) or fourfold lower dose (exp 3), and decreased if a 3–10-fold lower dose of MIF was administered over a longer time period of two weeks (Havel et al. 1996; Kroon et al. 2018). Thymus weights were consistently decreased, although not significant in the current study. As mentioned, this daily administration showing agonism contrasts with the continuous i.c.v. and twice a day high dose of MIF in that the latter two conditions produce in rodents disinhibition of the HPA axis in the circadian rise and following stress (van Haarst et al. 1996; Revsin et al. 2009).

These findings raise the question how the disinhibitory effect of acute MIF can abruptly change into an enduring inhibitory one upon repeated daily administration, particularly at doses in the range of therapeutic efficacy. A second, equally important question is, whether the current data contribute to understanding the apparent *reset* of the stress system achieved with MIF (Ratka et al. 1988; Hu et al. 2012). Various factors may contribute to this switch of GR antagonism to an apparent agonism, and ability to *reset*. These include (i) MIF kinetics: metabolism and penetration to GR feedback sites in brain and pituitary, (ii) dynamics of HPA-axis feedback regulation, (iii) differential signaling routes of MIF- and cortisol-occupied GR beyond HPA-axis regulation, and (iv) the brain MRs.

### MIF Kinetics

MIF is rapidly cleared because in rodents the antagonist is not bound to plasma proteins and rapidly metabolized, while in human blood MIF is extensively protected to degradation because of binding with high affinity to ɑ_1_ glycoprotein (Heikinheimo et al. 1987). We found that after a 50 mg/kg rat dose orally given during 5 days, after the fifth and last administration MIF is already depleted from the circulation in a 90-min post-ingestion interval, while low amounts of the antagonist and its metabolite RU42633 are retained in the brain for at least 3 h and in the same concentration range as corticosterone.

**Fig. 7:**
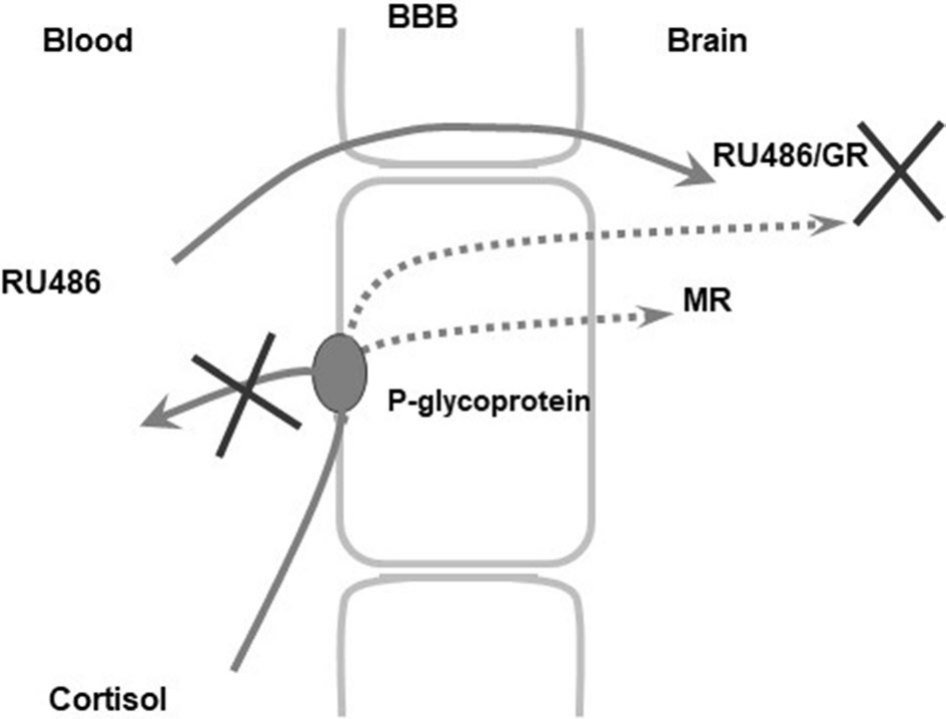
Hypothesized MIF-induced facilitation of cortisol brain uptake through inhibition of the efflux transporter P-glycoprotein at the blood–brain barrier. Under normal conditions, cortisol is hampered to enter the brain due to active outwards directed transport at the blood–brain barrier mediated by P-glycoprotein (Karssen et al. 2001). In the presence of MIF, this efflux is blocked facilitating the uptake of cortisol into the brain. The ensuing increased cortisol concentration will not lead to increased activation of GR, since this receptor is blocked by the high concentrations of MIF. However, increased activation of the MR is predicted to affect cognitive performance and neuroendocrine regulation. X indicates blockade of Pgp by MIF, which facilitates cortisol penetration through the blood–brain barrier and blockade of cortisol binding to GR. Dotted line is preferred cortisol route after Pgp blockade by MIF

The in vitro experiments using monolayers of pig kidney epithelial cells (LLC-PK1) stably expressing human MDR1 Pgp (Karssen et al. 2001) showed that MIF was capable of facilitating the transport of cortisol. We have previously shown that the uptake of dexamethasone and cortisol is hampered by Pgp at the blood–brain barrier in rodents and humans (Meijer et al. 1998; Karssen et al. 2001; Mason et al. 2012). MIF blocks Pgp, so cortisol cannot be bound and thus is not exported by the mdr transporter, hence a higher uptake and retention of cortisol. In an in vivo experiment, it was indeed demonstrated that a higher uptake of radioactivity in brain relative to blood occurred if ^3^H-cortisol is infused in animals pretreated with a high dose of MIF. The finding shows that repeated MIF could increase accumulation of the endogenous glucocorticoids in the brain of adrenally intact animals by blocking Pgp at the blood–brain barrier (Fig. 7).

In AtT 20 cells, MIF translocated the GR to the cell nucleus and induced DNA binding and MIF potentiated the cell nuclear translocation and DNA binding of corticosterone (Spiga et al. 2011). in vivo, MIF (20 mg/kg)-administered s.c. to ADX rats induced translocation and DNA binding of GR to nuclei of hippocampus 60 min following administration (van Eekelen et al. 1987; Spiga et al. 2011). The retention of the MIF–GR complex in pituitary nuclei was even more than twofold higher than in hippocampus. Pretreatment of the ADX rats with MIF did not inhibit but rather potentiated the nuclear retention of corticosterone (3 mg/kg) in hippocampal and pituitary nuclei; however, the effect was near trend level (*p* < 0,06) (Spiga et al. 2011). Whether this also occurs in vivo in intact animals with circulating corticosterone is not known.

In conclusion, the data show that in rodents MIF is rapidly cleared from the circulation but retained and bound to GR in hippocampal and pituitary cell nuclei and DNA. Most interestingly, MIF is a substrate for mdr transporters in the blood–brain barrier, and therefore in rodents the uptake and retention of exogenous cortisol is facilitated (Gruol et al. 1994; Lecureur et al. 1994; Gaillard et al. 1984; Karssen et al. 2001, 2005, 2004, 2002). Collectively, these data support the idea that at least in rodents very high doses of MIF are needed at the pituitary and brain level to overcome its rapid metabolism and the facilitated uptake and retention of cortisol.

### HPA-Axis Dynamics

One possibility to explain the sudden refractoriness to repeated MIF administration is that the pituitary ACTH stores are depleted. Such depletion is a common observation following ADX when after the initial surge in readily releasable ACTH minimum pituitary levels of irACTH are attained after 1–3 days. Meanwhile, synthesis of POMC is stepped up and a condition of several folds higher ACTH level is reached 1 week after ADX, which still can be further elevated by stress exposure (van Dijk et al. 1981; Jacobson et al. 1989). However, ACTH levels were increased during twice a day of MIF for 4 days (Revsin et al. 2009). It is therefore unlikely that ACTH exhaustion has occurred, but for a conclusive answer, however, the effect of MIF on pituitary and plasma ACTH needs to be studied still on a day-by-day basis.

An alternative explanation can be a recurrent negative feedback. After a single MIF challenge, corticosterone remained elevated for 16 h, while the genomic effects will persist even longer. Indeed, at 24 h, the 1xMIF-treated animals were capable of mounting a profound corticosterone response. Our study suggests a recurrent pattern of GR-mediated actions including negative feedback, which are transiently interrupted by daily application of the GR antagonist. We propose therefore that the HPA axis progressively adapts to this daily cycle of GR blockade and subsequent GR activation. Hence, during the seventh day of GR antagonist administration, the circadian corticosterone pattern may have become similar to that observed in control mice. Naturally, this assumption needs proof by measuring the effect of MIF on basal and stress induced on a day-by-day basis, and to compare this study with twice a day or continuous MIF administration thought to maintain disinhibition.

### MIF and Cortisol Signaling Via GR

The apparent agonism of MIF that is observed in some contexts may differ from the endogenous agonism via cortisol in several ways. First, it has been proposed that MIF–GR is much more potent in exerting transcriptional transrepression than transactivation activity (Heck et al. 1994). This effectively could mean that—once endogenous corticosterone levels are low—*part* of the GR-dependent negative feedback does get activated by MIF via transrepression (De Bosscher et al. 2003, 2016). This is consistent with the HPA-axis phenotype of the GR^dim/dim^ mouse, where transcription of the *Pomc* gene and memory performance are disrupted, because of its inability for GR homodimerization and transactivation, while the transrepression-dependent glucocorticoid negative feedback on ACTH release is mostly intact (Reichardt et al. 1998; Oitzl et al. 2001). Also, partial agonism of MIF via classical transactivation cannot be excluded in some cell types, and over time. For instance, in simple reporter gene assays, the extent of partial agonism could be doubled by overexpression of the SRC-1A coactivator (Meijer et al. 2005) that is relatively abundant in the core of the HPA axis (Meijer et al. 2000). Prolonged exposure to either continuous MIF, or alternating MIF and corticosterone in the mice studies, therefore has an unpredictable outcome, that may moreover develop over time, given that the expression of coactivators themselves may be regulated over time (Meijer et al. 2005).

### Mineralocorticoid Receptors

Irrespective of phasic or continuous GR blockade, corticosterone binding to the MR is not hampered by MIF (Reul et al. 1990). MR is known to mediate control over appraisal processes, behavioral reactivity to novel experiences, and the onset of HPA-axis activity (Joëls et al. 2008; de Kloet and Joëls 2017; Joëls and de Kloet 2017). The changes in corticosterone action via MR activation were reflected by the lower expression of hippocampal MR mRNA for the 1xMIF-group in all subregions, whereas expression was increased in the CA2 region of the hippocampus for 7xMIF-group. Interestingly, GR blockade after MIF treatment *per os* for three weeks, increased total hippocampal MR mRNA expression by 1.5 compared to controls (Bachmann et al. 2003). Therefore, it would be of interest to determine longitudinal effects of repeated GR blockade on MR function, particularly since previous studies have clearly shown that MR and GR interact in control of HPA-axis activity (Spencer et al. 1998).

Four days of MIF with the last administration given 90 min prior to the initial test promoted an active coping style (Wulsin et al. 2010). Acute MIF interfered with retention of this acquired immobility response, suggesting memory impairment (de Kloet et al. 1988). Chronic MIF, leaving MR active, improved memory performance (Oitzl et al. 1998). In line with this phenotype of a relative brain MR excess, studies with mouse mutants overexpressing MR in limbic forebrain revealed enhancement of memory (Ferguson and Sapolsky 2007; Lai et al. 2007; Kolber et al. 2008), perseveration of learned behavior (Harris et al. 2013), and reduction of anxiety (Rozeboom et al. 2007).

Previously, the pharmacological blockade of MR had also shown altered appraisal processes and selection of the appropriate behavioral response, i.e., search strategy (Oitzl and de Kloet 1992; Oitzl et al. 1994, 1997; Schwabe et al. 2010, 2013). In the current study, twenty-four-hours following repeated GR antagonism, mice used the serial search strategy more often, compared to controls. This strategy increases the likelihood that the animals will visit all possible escape routes that the circular hole board provides (during the exploration trial, all holes were closed). Indeed, the choice of applied strategy does affect performance in spatial learning trials (Dalm et al. 2000). In the current study, following 1xMIF, mice were initially slower to move away from the start center, but subsequently hyperactive on the circular hole board. This could indicate a change in the level of anxiety induced by previous GR antagonism. If so, then the effect is transient as repeated GR antagonism did not induce any of the above described features.

The elegant study by (Wulsin et al. 2010) revealed that one week treatment with a twentyfold lower dose of MIF i.p. (10 mg/kg rat) produced an attenuated HPA-axis response to a forced swim stressor. Interestingly, this course of MIF treatment also evoked a differential pattern of activation and inhibition of central inputs to the PVN. The ventral subiculum of the hippocampus and all regions of the medial frontal cortex showed enhanced stress-induced c-Fos activity after daily GR blockade, while the c-Fos response was reduced in other subregions of the hippocampus and in the amygdala. These data suggest that MIF enhanced inhibitory and suppressed excitatory inputs to the PVN that collectively may contribute to the downregulation of HPA-axis activity. To exert these effects, MIF likely may have recruited distinct cocktails of co-regulators of the GR in the corticosteroid target neurons of the limbic brain (Meijer et al. 2000; Meijer et al. 2005; Lachize et al. 2009; Ronacher et al. 2009; Zalachoras et al. 2013). In conclusion, as a result of GR blockade, the MR becomes relatively more activated. This combination of increased MR activity and blockage of GR may result in an altered function of limbic-frontocortical afferents and *reset* of neuroendocrine control of the HPA axis.

### Implications for Clinical Studies

One obvious question is how the current findings in the rodent may contribute to the clinical observations showing high-dose MIF efficacy. Recent studies referred to the need of circulating MIF levels exceeding 1637 ng/ml blood with a dosage of 1200 mg/day for 7 days in order to achieve the strongest association with treatment response in psychotic depression. This association in treatment response was followed by a weaker, but still significant association with increased basal ACTH and cortisol levels (Block et al. 2018), which lasted several weeks beyond the termination of MIF treatment. Before the conclusions on *reset* of the stress system from animal experiments can be extended, more data on stress responsivity in clinical studies are required, however.

The current data raise the possibility that MIF’s primary target is extrahypothalamic. A recent study demonstrated in rats that chronic MIF released from a 150 mg s.c. implanted pellet reduces (compulsatory) alcohol intake in dependent rats (Vendruscolo et al. 2012). These effects were reproduced by i.p. administration of 30–60 mg MIF at 90 min prior to alcohol intake as well as after 10–30 µg local administration bilaterally in the central amygdala. The data were reproduced in humans with MIF and the selective GR antagonist C-113176 (Vendruscolo et al. 2015). It was also found in rats that alcohol withdrawal and protracted abstinence produced changes in GR mRNA and bio-active phosphorylated GR rather than MR mRNA expression in the limbic-prefrontocortical and mesolimbic dopaminergic system. Using a systems biology approach, GR was identified as a master controller of downstream gene regulatory networks in these extrahypothalamic regions (Repunte-Canonigo et al. 2015).

When it concerns diseases precipitated by psychological stress, the ability of MIF and related compounds to readily re-establish the setpoint of the stress system is of prime importance. Such a *reset* would require restoration of an imbalance in MR/GR-mediated processes in the brain. A striking example is found in rats showing a deterioration of cellular and behavioral phenotype during chronic unpredictable stress, which is entirely restored with a single course of MIF (Hu et al. 2012). Transcriptome analysis (Datson et al. 2012) revealed that in laser-dissected hippocampal dentate gyrus of these chronically stressed animals, MIF treatment affected 107 genes, which were mostly different from the ones observed in the stressed group. We found that CREB-binding protein (CREB-BP) was normalized by MIF treatment to levels observed in control animals. Next to the GR, CREB-signaling, therefore, may play a central role in mediating the chronic stress effects on neurogenesis, LTP, and calcium currents in the dentate gyrus (Karst and Joëls 2003; van Gemert and Joëls 2006; Datson et al. 2012, 2013) and perhaps in other (meso)limbic-frontocortical regions as well.

In humans, bio-availability of MIF is extended because the steroid circulates bound to ɑ_1_ glycoprotein and therefore has a much longer half-life than in rodents, where this binding protein is absent (Heikinheimo et al. 1987). Moreover, in recent studies it was noted that although there is an obvious association with MIF’s action on the HPA axis, it cannot be excluded that the compound’s pharmacology is due to blockade of GRs in limbic-frontocortical and mesocortical dopaminergic circuits involved in mood, anxiety, reward, and motivation. This is reinforced by the MIF-induced change in stress circuit activation favoring inhibitory inputs to the PVN (Wulsin et al. 2010), the systems biology approach pointing to the GR in these regions as master controller (Repunte-Canonigo et al. 2015) and the MR:GR balance hypothesis (de Kloet et al. 2018). Although the required circulating MIF levels beyond 1637 ng MIF/ml blood certainly are an important first step, more data on HPA-axis regulation and extrahypothalamic effects as well as the role of (epi)genetic factors are needed to predict treatment response in psychotic depression.

### Perspectives

MIF (or Korlym®) is indicated for treatment of hyperglycemia during Cushing’s Syndrome and was found to cause a clinically significant metabolic improvement with less depressive symptoms, improved cognitive performance, and an increased quality of life (Fleseriu et al. 2012). This indication is based on the correction of glucocorticoid-induced glucose intolerance and diabetes mellitus (Beaudry et al. 2014; Teich et al. 2016; van den Heuvel et al. 2016; Moraitis et al. 2017; Kroon et al. 2018; Meijer et al. 2018).

Glucocorticoid action is of course needed for allocation of energy substrates to tissues in need during coordination of circadian events, immune and inflammatory defense reactions, stress coping, and adaptation (Picard et al. 2014; Hollis et al. 2015). If defense reactions essential for health exceed control by endogenous corticosterone and cortisol, they may become damaging themselves (Munck et al. 1984; Sapolsky et al. 2000). The exogenous (synthetic) glucocorticoids are very effective anti-inflammatory and immune suppressive agents. However, the adverse effects on energy metabolism, pituitary ACTH release, and brain function may become a serious concern. Important progress has been made by exploiting SGRM’s for identification of tissue and context-specific modulation of GR-mediated processes. This is important because the ‘golden bullet’ (Meijer et al. 2018) would be a compound that solely treats inflammatory and immune processes without side effects, or that solely restores deregulated metabolism, or that targets only central circuits underlying depression.

Potential new applications of the SGRM’s are being tested. For instance, C-108297 (3,5 mg/mouse) for 4 days given to Wobbler mice, an animal model of human amyotrophic lateral sclerosis (ALS), normalizes indices for neurogenesis and facilitates recovery from a pro-inflammatory phenotype in hippocampus (Meyer et al. 2014). The selective GR antagonist C-113176 displayed a similar protective pharmacology in the Wobbler mice, and additionally prevented spinal cord pathology, while displaying anti-inflammatory and anti-glutamatergic activity (Meyer et al. 2018).

MIF (30 mg/kg rat ip), and the much more effective C-108297 (20 mg/kg) and C-113176 (10 mg/kg) at a dose of 20 and 10 mg/kg, respectively, twice a day for 5 days, all reversed the precipitation of Alzheimer pathology and cognitive impairment in an animal model generated by hippocampal amyloid-β_25−35_ administration, while normalizing plasma corticosterone levels (Pineau et al. 2016). Another interesting new ligand is C-118335, a mixed GR modulator/MR antagonist (100 mg/ kg male rat), which displayed clearly GR agonistic activity in gene regulation and HPA-axis suppression after a single s.c. injection, but blocked memory storage (Atucha et al. 2015), while being inactive on coping style (Nguyen et al. 2017, 2018). Hence, more selective GR modulators will be important for targeted treatment.

## Concluding remarks

The *reset* of stress system activity by MIF could be due to the following factors: (1) The detrimental effects of high corticosteroid concentrations via GR activation are prevented by GR antagonism. This approach has obvious benefit during continuous or high-frequency blockade of the GR. (2) The high circulating corticosterone concentrations may have caused a long-lasting rebound suppression of HPA-axis activity, particularly since MIF is rapidly cleared. (3) MIF may become an agonist in transrepression (Heck et al. 1994) and promote cell- and context-dependent recruitment of coactivators and co-repressors in a cell-specific fashion in brain stress circuitry (Meijer et al. 2018; Zalachoras et al. 2013, 2016). (4) Enhanced brain MR activation may become prominent relative to the MIF-modulated GR. The altered MR:GR balance could be part of a compensatory mechanism producing altered patterns of inhibitory and excitatory circuits underlying *reset* of stress system activity (De Kloet et al. 1998; de Kloet et al. 2005, 2018; Sousa 2016; Wulsin et al. 2010). This includes the role of MR and GR as homo- or heterodimers in binding to GRE’s, transcription factors, and co-regulators (Mifsud and Reul 2016; Van Weert et al. 2017; Matosin et al. 2018; Meijer et al. 2018) as well as their rapid non-genomic actions (Di et al. 2003; Karst et al. 2005, 2010; Groeneweg et al. 2012; Hill and Tasker 2012).

Chronic stress models of depression display glucocorticoid resistance caused by imbalance in the afferent limbic-prefrontocortical pathways innervating the hypothalamic PVN and mesolimbic dopaminergic system (Ulrich-Lai and Herman 2009). The striking finding with MIF and related compounds is that the high doses required to affect pituitary and brain regulation of the HPA axis seem capable of readily *resetting* stress system activity. It is a great challenge to discover if *reset* of stress system activity is the cause or the consequence of MIF’s efficacy in the treatment of stress-related neuropsychiatric disorders.
